# Supplementary material for: Chained Structure of Dimeric F1-like ATPase in Mycoplasma mobile Gliding Machinery
Source: mBio. 2021 Jul 20;12(4):e01414-21. doi: 10.1128/mBio.01414-21 (PMC8406192; doi:10.1128/mBio.01414-21)
Supplement: FIG S1 [file mbio.01414-21-sf001.pdf]

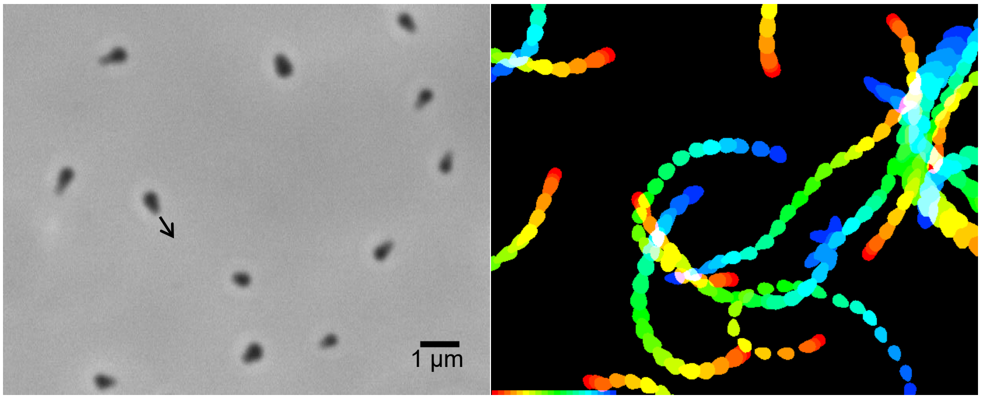

FIG S1 Gliding of *M. mobile* cells. Optical microscopy of cells (left) and trajectories of gliding cells (right). All cells are gliding in the direction of tapered end as indicated by a black arrow. For trajectories, video frames of every 0.2 s were colored differently from red to blue, and stacked for 4 s.
