# Supplementary material for: Chained Structure of Dimeric F1-like ATPase in Mycoplasma mobile Gliding Machinery
Source: mBio. 2021 Jul 20;12(4):e01414-21. doi: 10.1128/mBio.01414-21 (PMC8406192; doi:10.1128/mBio.01414-21)
Supplement: FIG S2 [file mbio.01414-21-sf002.pdf]

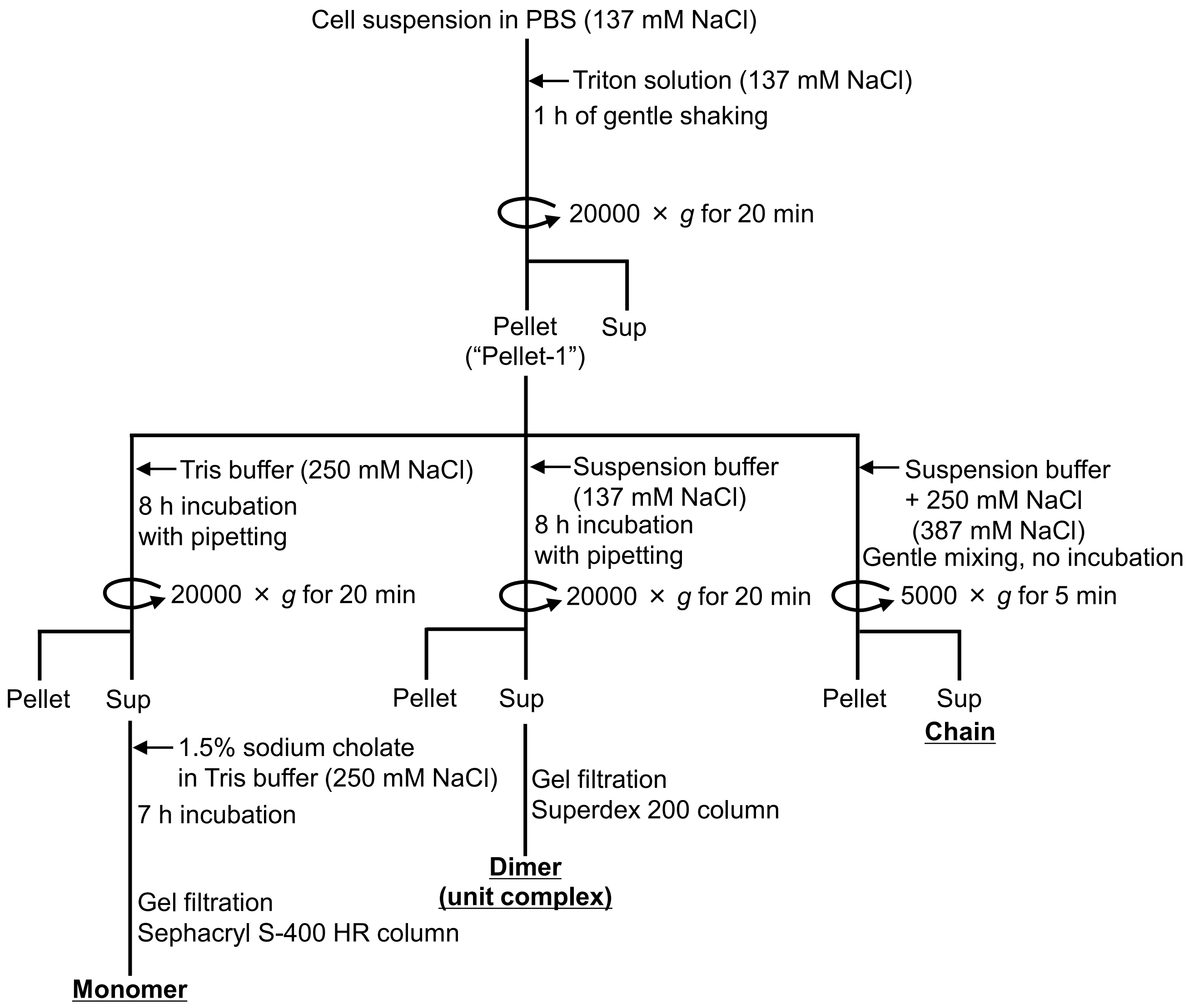

FIG S2 Isolation procedure for three fractions. Each fraction was obtained from Pellet-1 fraction. The adjusted concentrations of NaCl in the solution are indicated in parentheses. Supernatant is abbreviated as "Sup". See Materials and Methods for details.
