# Supplementary material for: Chained Structure of Dimeric F1-like ATPase in Mycoplasma mobile Gliding Machinery
Source: mBio. 2021 Jul 20;12(4):e01414-21. doi: 10.1128/mBio.01414-21 (PMC8406192; doi:10.1128/mBio.01414-21)
Supplement: FIG S3 [file mbio.01414-21-sf003.pdf]

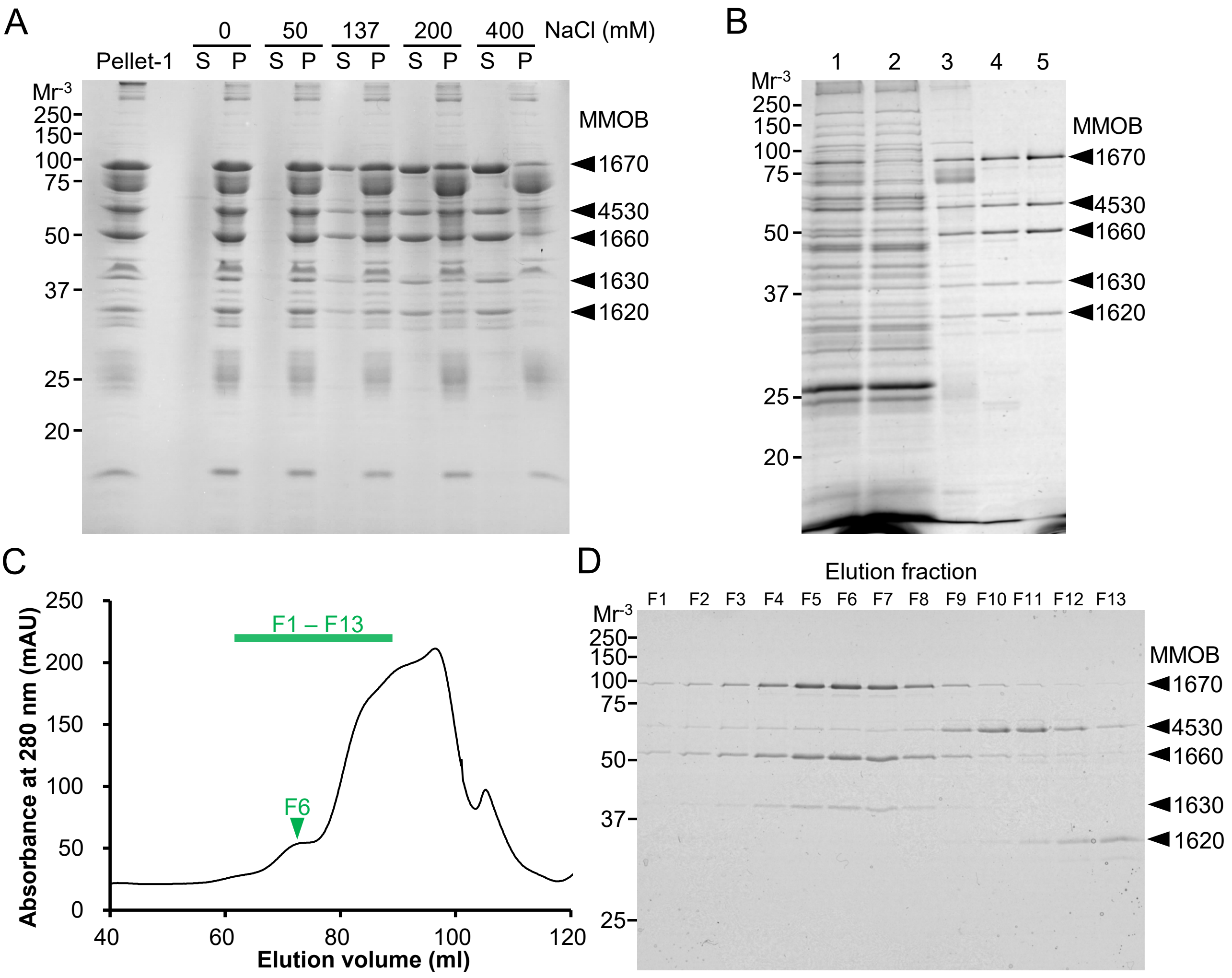

FIG S3 Isolation profile of Dimer (unit complex) and Monomer fractions. (A) Solubility of the chain components. Pellet-1 fraction was treated with buffers containing the specified concentrations of NaCl and centrifuged. Pellet-1 fraction, the supernatants (S) and the pellets (P) were analyzed by SDS-12.5% PAGE. See Materials and Methods for details. (B) Isolation of Dimer (unit complex). The fractions of each step were subjected to SDS-12.5% PAGE gel and stained with CBB. Lane 1, lysate of *M. mobile* cells; lane 2, Triton-soluble fraction; lane 3, Triton-insoluble (Pellet-1) fraction; lane 4, supernatant after incubation in suspension buffer containing 137 mM NaCl; lane 5, peak fraction of Superdex 200 gel filtration chromatography. The bands of Dimer components are marked by black triangles. (C) Gel filtration profile in Monomer isolation using a Sephacryl S-400 HR column. The small peak is marked by a green triangle. (D) The F1–F13 fractions in the gel filtration indicated by the green line in panel C were subjected to SDS-12.5% PAGE and stained with CBB. F6, which corresponds to the small peak in the gel filtration, was used for further analyses as Monomer fraction. Bands of Dimer components are marked by black triangles. For panels (A, B, D), Focused proteins were identified by PMF, and molecular masses are shown on the left.
