# Supplementary material for: Chained Structure of Dimeric F1-like ATPase in Mycoplasma mobile Gliding Machinery
Source: mBio. 2021 Jul 20;12(4):e01414-21. doi: 10.1128/mBio.01414-21 (PMC8406192; doi:10.1128/mBio.01414-21)
Supplement: FIG S4 [file mbio.01414-21-sf004.pdf]

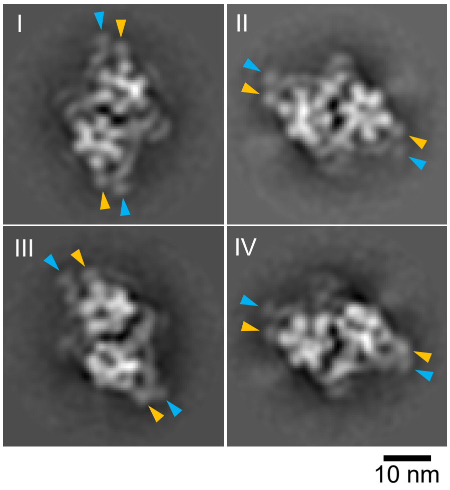

FIG S4 Two-dimensional averaged images of Dimer obtained by negative-staining EM. Four classes of clear particle images from 20 classes are represented. These images have common features as represented long and short protrusions marked by light blue and orange triangles, respectively. As mentioned in Fig. 3C, the images were mirrored to uniform handedness.
