# Supplementary material for: Chained Structure of Dimeric F1-like ATPase in Mycoplasma mobile Gliding Machinery
Source: mBio. 2021 Jul 20;12(4):e01414-21. doi: 10.1128/mBio.01414-21 (PMC8406192; doi:10.1128/mBio.01414-21)
Supplement: FIG S5 [file mbio.01414-21-sf005.pdf]

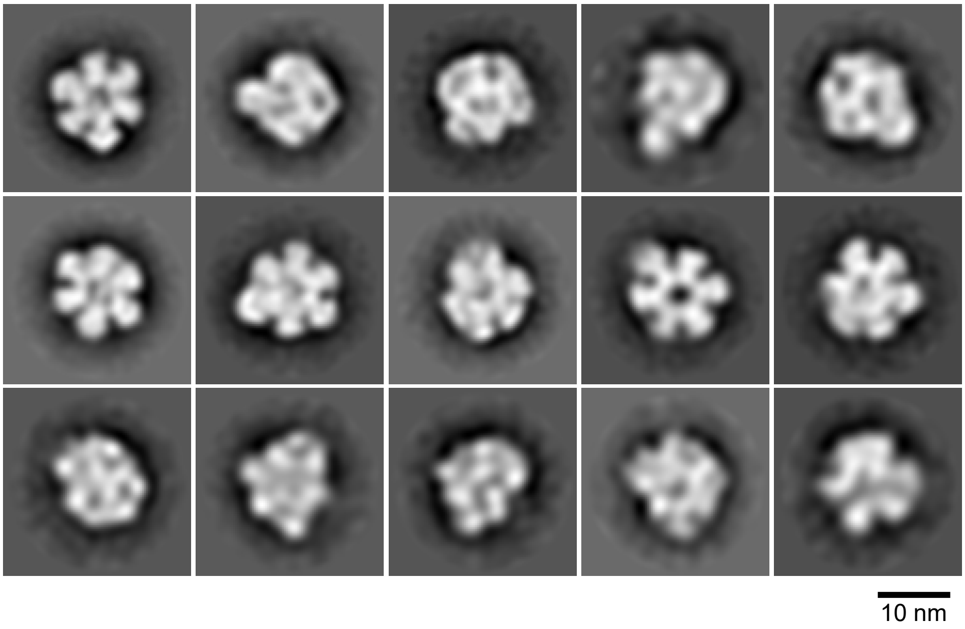

FIG S5 Two-dimensional averaged images of globular complex in Monomer fraction. Fifteen classes of clear particle images from 50 classes are represented.
