# Supplementary material for: Chained Structure of Dimeric F1-like ATPase in Mycoplasma mobile Gliding Machinery
Source: mBio. 2021 Jul 20;12(4):e01414-21. doi: 10.1128/mBio.01414-21 (PMC8406192; doi:10.1128/mBio.01414-21)
Supplement: FIG S6 [file mbio.01414-21-sf006.pdf]

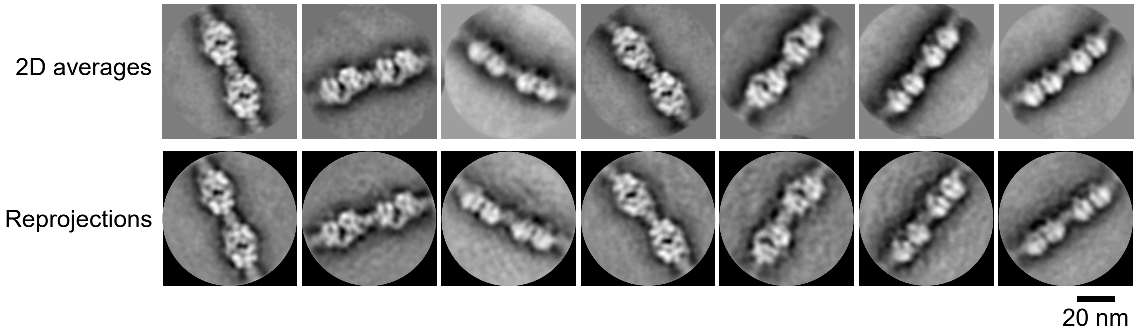

FIG S6 Reprojection images of Chain. Two-dimensional averaged images (upper) and the corresponding reprojection images (lower) calculated from the 3D map of Chain are compared.
